# Relationship between tumor microbiota transcriptional activity and gene expression in breast cancer

**DOI:** 10.1186/s12885-023-10726-4

**Published:** 2023-03-16

**Authors:** Enuo Liu, Fan Zhang, Tiansheng Xu, Luyi Ye, Sean Si Qian Ma, Zai-Si Ji

**Affiliations:** 1 NHC Key Laboratory of Reproduction Regulation, Shanghai Institute for Biomedical and Pharmaceutical Technologies), Fudan University, 2140 Xietu road, 200032 Shanghai, China; 2 College of Food Sciences and Technology, Shanghai Ocean University, 999 Hucheng Road, Shanghai, China; 3 Division of Research and Development, Meiji Co., Ltd, 1-29-1 Nanakuni, 192-0919 Tokyo Hachiouji, Japan; 4 Shanghai OE Biotech Co., Ltd, 1188 Lianhang road, 201114 Shanghai, China

**Keywords:** Tumor microbiota, ER-negative breast cancer, Carcinogenesis, *Lactobacillus*, *Bacteroides*

## Abstract

**Background:**

A few studies have reported the distribution of the microbiota in breast cancer tissues, but few reports have compared the microbiota in different subtypes of breast cancer tissue. Moreover, no study has reported on the relationship between the microbiota and gene expression in breast tumor.

**Methods:**

Sections of formalin-fixed paraffin-embedded (FFPE) tissue were prepared from the breast tumors of 70 patients and were subjected to microarray analysis to identify gene expression profiles. The same total RNA samples were also used to analyze the microbiota activity in tumor tissues by performing 16 S rRNA sequencing and internal transcribed spacer (ITS) sequencing of reverse transcript cDNA with Illumina Miseq. Pearson’s correlation coefficient was used for calculating the correlation between microbial relative activity and gene expression.

**Results:**

The microbiota transcriptional activity of 70 FFPE samples mainly consisted of the phyla *Bacteroidetes, Firmicutes* and Proteobacteria. *Prevotella_9, Bacteroides* and Alloprevotella were the most active genera in ER+/HER2-, ER+/HER2 + and ER-/HER2 + tumors, while triple-negative samples exhibited a higher activity of *Lactobacillus*. In ER-negative samples (triple-negative and ER-/HER2+), 479 genes, including the breast carcinogenesis genes phospholipase A2, histone cluster 2, Crk-like, and cyclin D1, were significantly positive associated with the activity of *Lactobacillus*.

**Conclusion:**

This was the first study to clarify an association between the breast tumor microbiota transcriptional activity and the expression of carcinogenesis genes in ER-negative breast cancer. Changes in the microbiota of breast tissue induced by external factors might be one of the key causes of ER negative breast cancer.

**Supplementary Information:**

The online version contains supplementary material available at 10.1186/s12885-023-10726-4.

## Introduction

Breast cancer is a hormonally driven cancer especially for estrogen receptor (ER) positive breast cancer [1]. For patients with ER-positive breast cancer, hormonal therapy such as treated with tamoxifen for 5 years reduces the annual breast cancer death rate by 31% [2], and tamoxifen therapy for 10 years can halve breast cancer mortality during the second decade after diagnosis [3]. Conversely, such hormonal therapy has little or no effect on recurrence or mortality in patients with ER-negative breast cancer [4]. The majority of prognostic markers, which are overexpressed in patients with good prognosis in ER-negative breast cancer including HER2-overexpressing breast cancer and triple-negative breast cancer [5], associated with the activation of complement and immune response pathways [6]. Immunotherapies have been used to treat ER-negative breast cancer [7].

Several studies reported unique microbial signatures in tissue in most major types of cancer (breast, prostate, lung, ovary, pancreas, bone, melanoma and brain tumors), and breast cancer has a particularly rich and diverse microbiota [8, 9]. In samples collected from outside of the marginal zone positioned approximately 5 cm away from the tumor’s edge in Italian subjects, researchers identified more similarities than differences between breast tumors and adjacent normal tissues [10]. The most active taxa in breast tissue samples obtained from Canadian subjects, which were collected outside the marginal zone 5 cm from the tumor, were *Bacillus* (11.4%), *Acinetobacter* (10.0%), *Enterobacteriaceae* (8.3%), *Pseudomonas* (6.5%), *Staphylococcus* (6.5%), *Propionibacterium* (5.8%), *Comamonadaceae* (5.7%), *Gammaproteobacteria* (5.0%), and *Prevotella* (5.0%) [11]. In breast tissue samples collected in Ireland (taken at least 5 cm from the primary tumor site), the most active taxa were *Enterobacteriaceae* (30.8%), *Staphylococcus* (12.7%), *Listeria welshimeri* (12.1%), *Propionibacterium* (10.1%), and *Pseudomonas* (5.3%) [11], indicated that there might be significant differences among ethnic groups. A higher relative abundance of *Bacillus* (*Firmicutes*), *Enterobacteriaceae* (*Proteobacteria*) and *Staphylococcus* (*Firmicutes*) was reported in Canadian women with breast cancer than in those without breast cancer [12]. Conversely, an increased relative abundance of gram-positive organisms including *Corynebacterium* (*Actinobacteria*), *Staphylococcus* (*Firmicutes*), *Actinomyces* (*Actinobacteria*), and *Propionibacteriaceae* (*Actinobacteria*), and a decreased relative abundance of *Methylobacterium* (*Proteobacteria*) were reported in American women with breast cancer compared with those in American women without breast cancer [13].

Although the distinct microbial characteristics of breast tumor tissues have been compared with those of normal adjacent tissue, breast skin tissue, breast skin swabs, and buccal swabs [14], the association of the microbial community with gene expression has not been reported, and few analysis based on the ER, progesterone receptor (PR) and human epidermal growth factor receptor-2 (HER2) status has been described. In this study, we focused on the microbiota activity of subtype breast cancer tissues by 16s RNA / ITS (Internal transcribed spacer) sequencing of cDNA reverse transcripted from RNA, and its association with gene expression obtained by RNA microarray hybridization. The results could provide not only the microbiota of breast cancer subtype, but also the relationship between microbiota and gene expression which might contribute new insights into the clinical treatment of breast cancer.

## Materials and methods

### Study design and sample collection

Seventy formalin-fixed paraffin-embedded (FFPE) samples were collected in Huangpu District Central Hospital of Shanghai, China. The samples were prepared from fresh ductual tumors in women with breast cancer (age, 43–76 years) undergoing breast surgery at the operation room of the same hospital. The tumor tissues were preserved in formalin and transferred to the laboratory immediately. FFPE processing was performed in the sterile clean room using sterilized materials and new open reagents. FFPE samples were divided into four groups based on the ER, PR and HER2 positive (+) or negative (-) status, including 7 samples from ER+/HER2- patients, 48 samples from ER+/HER2 + types, 8 samples from ER-/HER2 + patients and 7 samples from triple-negative patients confirmed by immunohistochemistry.

### RNA extraction, microarray hybridization, and data analysis

A RecoverAll™ Total Nucleic Acid Isolation Kit (Ambion, AM1975) was used to extract total RNA from 10-µm FFPE tumor tissues and two environmental controls according to the manufacturer’s protocol. As environmental controls, the blank digestion buffer added protease was processed in parallel with the tumor tissue samples. The process was performed in clean room mainly involved deparaffinization, protease digestion, nucleic acid isolation, DNase digestion, and final purification. RNA purity and quantification were evaluated using a NanoDrop 2000 spectrophotometer (Thermo Fisher Scientific, USA). RNA integrity was assessed using an Agilent 2100 Bioanalyzer (Agilent Technologies, Santa Clara, CA, USA).

Gene expression profiles were investigated using an Affymetrix Human Clariom™D Assay (OE Biotechnology Co., Ltd., Shanghai, China). Sample labeling, microarray hybridization, and washing were performed according to the manufacturer’s standard protocols. Briefly, total RNA was transcribed into double-stranded cDNA and purified. Next, second-cycle cDNAs were used for fragmentation and biotin labeling. Labeled cDNA samples were hybridized to microarrays. After washing and staining, the microarrays were scanned using an Affymetrix Scanner 3000 (Affymetrix).

For microarray analysis, Affymetrix GeneChip Command Console (version 4.0, Affymetrix) software was used to extract raw data. Next, Expression Console (version1.3.1, Affymetrix) software was used for RMA normalization for gene analysis. Differentially expressed genes were then identified using Genespring software (version 13.1; Agilent Technologies). The difference in expression between each group of microarray samples was expressed as the fold-change. A heatmap generated using the clustermap function of the Seaborn package in Python was used to illustrate gene expression among samples. GO analysis and KEGG analysis were applied to determine the roles of these differentially expressed mRNAs played in these GO terms or pathways. The microarray data have been uploaded to the GEO database under the accession codes GSE183231 and GSE185439.

### 16 S rRNA sequencing, bacterial diversity, and taxonomic analysis

After extraction and quality control, 0.5 µg of RNA from each sample was used for reverse transcription (10 µl), and the cDNA was diluted to 100 µl for subsequent 16 S library construction. PCR amplification of the V3-V4 hypervariable regions of the bacterial 16 S rRNA was performed in a 25 µl reaction volume using universal primer pairs (343 F: 5′-TACGGRAGGCAGCAG-3′; 798R: 5′-AGGGTATCTAATCCT-3′). The reverse primer contained a sample barcode, and both primers were linked to an Illumina sequencing adapter (Illumina, San Diego, CA, USA). The amplicon products were purified using Agencourt AMPure XP beads (Beckman Coulter Co., USA) and quantified using a Qubit dsDNA assay kit. Sequencing was performed on an Illumina Miseq with two paired-end read cycles of 300 bases each. The 16 S rRNA sequencing data have been uploaded to the GenBank Sequence Read Archive under accession code PRJNA769523.

Paired-end reads were preprocessed using Trimmomatic software [15] to detect and remove ambiguous bases (N) in the data quality control step. Clean reads were subjected to primer sequence removal and clustering to generate operational taxonomic units (OTUs) using VSEARCH software with a similarity cutoff of 97% [16]. The representative read of each OTU was selected using the QIIME package, and then all representative reads were annotated using the Silva database (Version 123) and the RDP classifier [17]. The microbial richness and diversity of breast cancer samples was estimated using indices of alpha diversity including Good’s coverage, the Chao1 index (community richness) [18] and Shannon index (diversity) [19]. The UniFrac distance matrix generated using QIIME software was used for unweighted UniFrac nonmetric multidimensional scaling (NMDS) to display bacterial beta diversity.

### Internal transcribed spacer (ITS) sequencing, fungal diversity, and taxonomic analysis

For fungal diversity analysis, the ITS1 variable regions were amplified using the universal primer pairs (ITS1F: 5′-CTTGGTCATTTAGAGGAAGTA-3′; ITS2: 5′-GCTGCGTTCTTCATCGATGC-3′) after reverse transcription. After PCR product purification and library quantification, sequencing was performed on an Illumina MiSeq with two paired-end read cycles of 300 bases each. (Illumina Inc., San Diego, CA). The ITS sequencing data have been uploaded to the GenBank Sequence Read Archive under accession code PRJNA769523.

Data quality control, OTU generation, and representative sequence selection were performed using the same methods described for 16 S rRNA sequencing. All representative reads were annotated and searched against the UNITE and NCBI databases (ITS rDNA) using BLAST [20]. Then, the fungal diversity including alpha diversity (Good’s coverage, Chao1 index and Shannon index) and beta diversity (UniFrac distance for NMDS) was used to assess the distribution of fungi in each sample and the differences between groups. R package was used to analyze the significant differences among four groups using Analysis of Variance (ANOVA) statistical test.

### Correlation analysis

The correlation between gene expression and the relative activity of bacteria was calculated using Pearson’s correlation coefficient. The threshold set for a significant correlation was r > 0.7 and p < 0.05.

## Results

### Data of surgically treated patients

The clinical and pathological features of the 70 patients are summarized in Table 1. Compared to the triple-negative group, the other three groups had no significant difference for age at surgery (*p* < 0.05). There also was no significant difference among the four groups regarding tumor size, lymph nodes, and tumor grade by chi-square test.

**Table 1:** Clinical and pathological features of 70 breast cancer patients in surgery

	ER+/HER2-	ER+/HER2+	ER-/HER2+	Triple -negative	*P* value
**subjects (n)**	7	48	8	7	
**age at surgery (year)**	48.9 ± 10.1	54.0 ± 8.4	53.9 ± 10.6	59.0 ± 11.5	
*P* value	0.105 *	0.167 *	0.386 *		
**ER status (IHC)**	+	+	-	-	
**PR status (IHC)**	+	+	-	-	
**HER2 status (IHC)**	-	+	+	-	
**lymph node**					
Positive (n)	5 (71%)	24 (50%)	5 (63%)	5 (71%)	0.497#
Negative (n)	2 (29%)	24 (50%)	3 (38%)	2 (29%)	
**Tumor size**					0.943#
> 20 mm (n)	3 (43%)	21 (44%)	4 (50%)	4 (57%)	
≦ 20 mm (n)	4 (57%)	27 (56%)	4 (50%)	3 (43%)	
**Histological grade**					
grade 1	0 (0%)	7 (15%)	1 (13%)	1 (14%)	0.153#
grade 2	4 (57%)	30 (63%)	2 (25%)	3 (43%)	
grade 3	3 (43%)	11 (23%)	5 (63%)	3 (43%)	

ER+: ER-positive > 10% tumor cells; ER-: ER-positive < 10% tumor cells. PR+: PR positive > 10% tumor cells; PR-: PR-positive < 10% tumor cells. HER2+: HER2 positive > 10% tumor cells; HER2-: HER2 positive < 10% tumor cells; Data are means ± standard deviations or presented as number of patients (%). Percentages are calculated from the total number of patients with known values. * Student’s *t* test; *p* value: ER+/HER2- versus triple-negative; or ER+/HER2 + versus triple-negative; or ER-/HER2 + versus triple-negative; # chi-square test

### Bacteria in four types of breast tumor

**Fig. 1:**
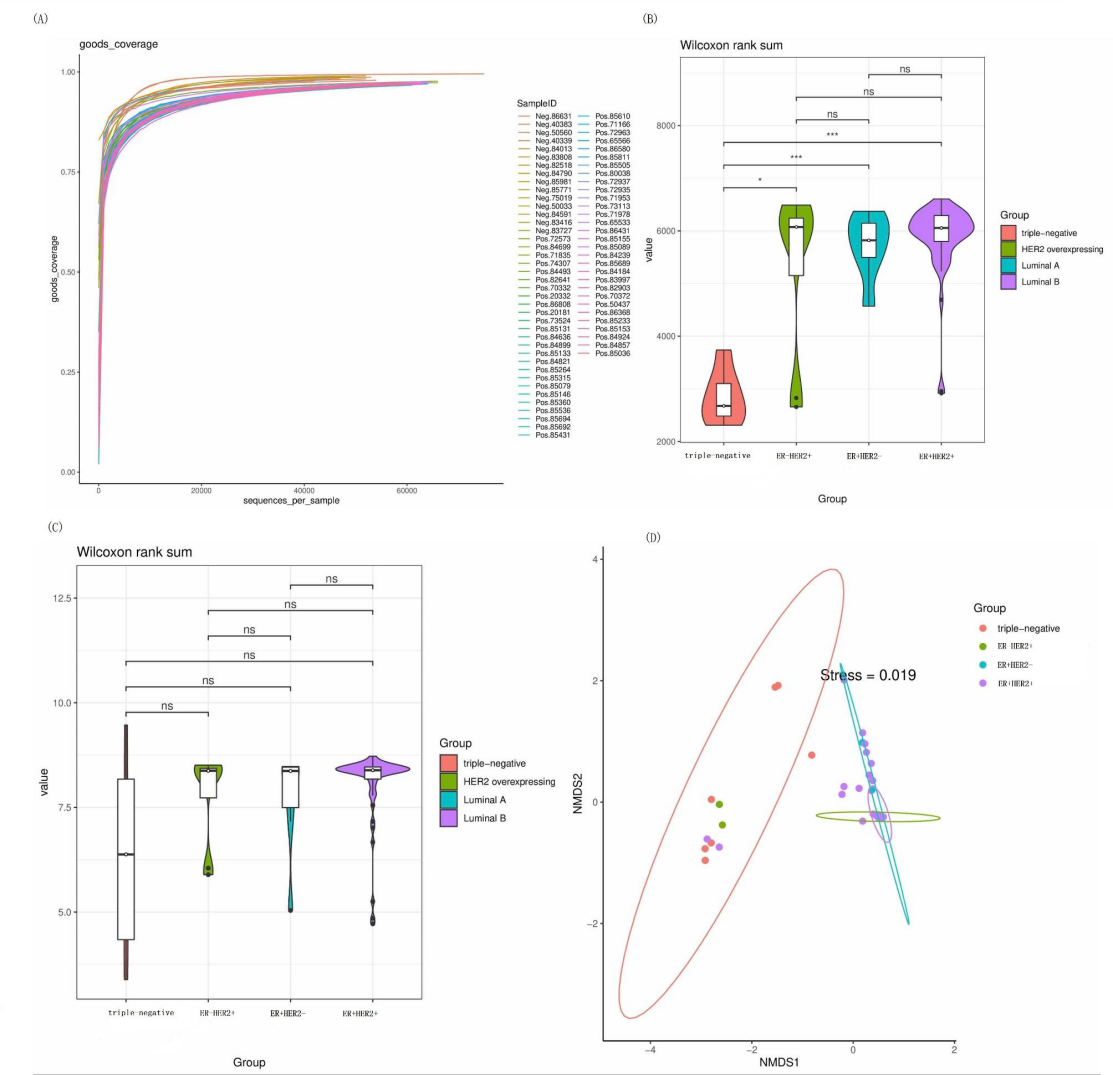
Analysis of bacterial profiles in four groups. (A) Good’s coverage for the four groups; (B) comparison of bacterial richness (Chao 1) among the four groups; (C) comparison of bacterial diversity (Shannon) among the four groups; (D) analysis of beta diversity (NMDS) among the four groups. Wilcoxon rank sum test was used for comparisons. * *p* value < 0.05, ** *p* value < 0.01, *** *p* value < 0.001

16 S rRNA gene amplicon sequencing of the V3-V4 hypervariable regions was performed using cDNA samples reverse transcript from total RNA extracted from 70 FFPE samples. In total, 4,846,572 high-quality sequences of the 16 S rRNA gene in 70 samples were obtained by high-throughput DNA sequencing, and 23,032 OTUs were obtained. Good’s coverage exceeded 95.5% for the four groups, indicating that the sequencing depth was sufficient for tissue microbiota investigation in patients with breast cancer (Fig. 1A). According to the alpha diversity, triple-negative tumors exhibited lower richness (Chao index, Fig. 1B) for the lower 8018 OTUs detected than other three groups 12,658 OTUs (ER-/HER2+), 12,721 OTUs (ER+/HER2-), 19,974 OTUs (ER+/HER2+), respectively. However, there was no significant difference in the diversity indicated by the Shannon index among the four groups (Fig. 1C). The NMDS analysis (Fig. 1D) revealed differences in the tissue microbiota composition of triple-negative and other three groups tumors. On the other hand, the two environmental controls contained completely different microbiota abundance compared to the 70 tumor samples (supplementary file 1).

**Fig. 2:**
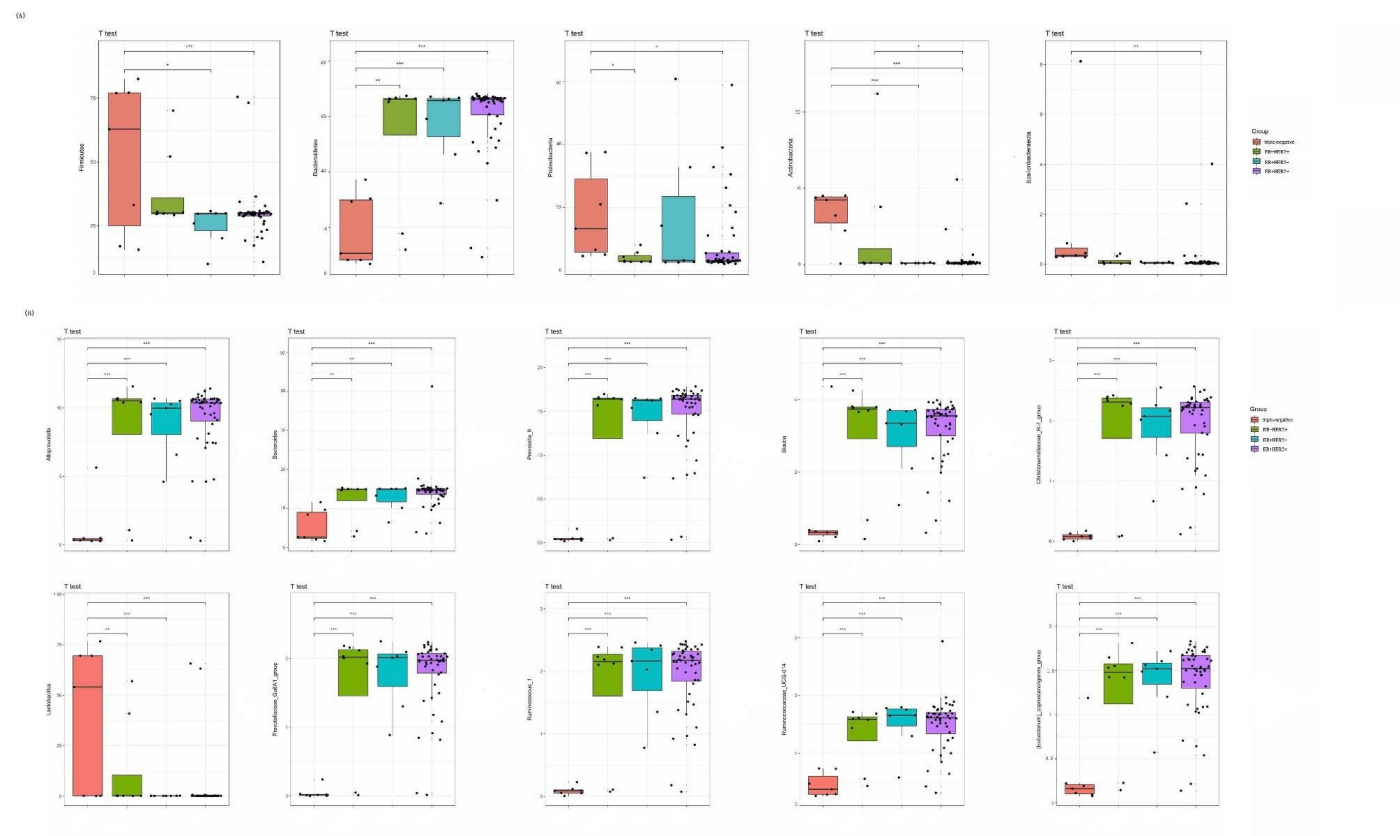
Comparison of the microbial relative activity (%) among the four groups (A) Box plots of the top 5 phyla; (B) box plots of the top 10 genera; The black line inside the box represents the median. The vertical line represents the lowest datum still within 1.5 interquartile range (IQR) of the lower quartile and the highest datum still within 1.5 IQR of the upper quartile. Student’s t test was used for comparisons. * *p* value < 0.05, ** *p* value < 0.01, *** *p* value < 0.001

A total of 35 phyla were found and the relative activity exceeded 85% for the three major bacterial phyla *Bacteroidetes*, *Firmicutes*, and *Proteobacteria* (Table 2). The triple-negative group displayed a significantly lower relative activity of *Bacteroidetes* than other three groups (p < 0.01 or p < 0.001), and a significantly higher relative activity of *Firmicutes* than ER+/HER2- (p < 0.05) and ER+/HER2 + group (p < 0.001) (Fig. 2A). At the genus level, the triple-negative group exhibited a significantly higher relative activity of *Lactobacillus* (p < 0.01 or p < 0.001), and significantly lower relative activity of *Prevotella_9*, *Allorevotella* and *Bacteroides* (p < 0.01 or p < 0.001) than other three groups (Fig. 2B). *Prevotella_9*, *Allorevotella* and *Bacteroides* are anaerobes belongings to Bacteroidetes, whereas *Lactobacillus* consists of facultative anaerobes belongings to Firmicutes.

**Table 2:** Mean relative activity of the top 5 bacteria phyla and top 10 genuses in ER-/HER2 + and other three groups

	Mean of relative activity (%)			
Taxa-phylum	ER+/HER2-	ER+/HER2+	ER-/HER2+	triple-negative
*Firmicutes*	25.21	30.25	37.69	52.20
*Bacteroidetes*	57.21	60.53	53.55	18.65
*Proteobacteria*	17.04	8.07	4.10	17.96
*Actinobacteria*	0.21	0.57	3.88	6.61
*Epsilonbacteraeota*	0.06	0.20	0.13	1.52
Taxa-genus				
*Prevotella_9*	14.40	14.72	12.42	0.54
*Lactobacillus*	0.20	2.89	12.38	38.62
*Bacteroides*	12.88	14.24	12.11	5.54
*Alloprevotella*	8.87	9.25	8.21	1.12
*Blautia*	2.99	3.19	2.98	0.87
*Christensenellaceae_R-7_group*	1.88	1.94	1.78	0.08
*Ruminococcus_1*	1.93	1.95	1.70	0.09
*Prevotellaceae_Ga6A1_group*	1.78	1.78	1.56	0.05
*Ruminococcaceae_UCG-014*	1.50	1.50	1.33	0.45
*[Eubacterium]_coprostanoligenes_group*	1.38	1.40	1.22	0.29

### Fungal diversity analysis in four types of breast tumor

ITS1 variable region sequencing was performed on cDNA samples reverse transcript from total RNA extracted from 70 FFPE samples. In total, 3,966,334 high-quality sequences of ITS1 genes were obtained by high-throughput DNA sequencing, and 3,441 OTUs were obtained. Good’s coverage exceeded 99.6% for the four groups (Supplementary file 2 A), indicating that the sequencing depth was sufficient for tissue microbiota investigation in patients with breast cancer. According to the alpha diversity (Supplementary file 2B-C), four groups displayed similar richness (Chao1 index) and ER-/HER2 + group displayed higher diversity (Shannon index, p < 0.01)), indicating the better homogeneity of OUT sequences in ER-/HER2 + group. NMDS analysis (Supplementary file 2D) revealed that the fungal composition was similar among triple-negative, ER-/HER2+, ER+/HER2- and ER+/HER2 + tumors.

Regarding the fungal community, the relative activity of phylum *Ascomycota* exceeded 30% in all of the groups (Supplementary file 3). The groups exhibited a similar relative activity (FDR p > 0.05) at the top 4 phyla (mean relative activity > 0.01) including *Ascomycota, Basidiomycota*, *Rozellomcota*, and *Mortierellomycota* (Supplementary file 3),.and also exhibited a similar relative activity (FDR p > 0.05) at class, order, family and genus using ANOVA statistical test. On the other hand, the two environmental controls failed in amplification step, indicating no fungi existed (supplementary file 1).

### Microarray analysis of gene expression in ER-negative and ER-positive breast tumors

Gene expression profiling was performed on RNA samples obtained from 70 breast tumors using the Affymetrix Human Clariom™D Assay containing 135,750 probe sets including 18,858 Entrez gene RNAs and 66,845 lincRNAs. Changes in the expression of all the RNAs corresponding to the probe sets on the microarray were depicted in Supplementary file 4 A. Using the arbitrarily chosen criteria of > 1.5-fold change in expression and p < 0.05, 478 transcripts were determined to be differentially expressed between ER-negative (triple-negative and ER-/HER2+) and ER-positive (ER+/HER2- and ER+/HER2+) tumors. In total, 440 genes were upregulated in ER-positive tumors including 149 coding genes, 121 noncoding genes, and 170 other genes (Supplementary file 4B). In particular, the expression of arginine and glutamate rich 1, a MED1-interacting protein required for estrogen-dependent gene transcription and breast cancer cell growth [21], was 4.9-fold higher in ER-negative tumors than ER-positive tumors (p < 0.001, FDR p = 0.0267). Moreover, 38 genes had lower expression in ER-negative tumors, including 8 coding genes, 21 noncoding genes, and 9 other genes (Supplementary file 4B).

As revealed by Gene Ontology (GO) enrichment analysis between ER-negative and ER-positive groups, the DEGs were associated with 160 GO terms including 102 in the category of “biological process”, 32 in the category of “cellular component” and 26 in the category of “molecular function”. The top 20 GO terms are presented in Supplementary file 4 C. Kyoto Encyclopedia of Genes and Genomes (KEGG) pathway enrichment analysis revealed differentially expressed genes in six pathways such as “One carbon pool by folate” (two genes, p = 0.003, Supplementary file 5) [22].

### Correlation analysis between gene expression and the activity of *Lactobacillus* in ER-negative tumors

A strong correlation between gene expression and the relative activity of *Lactobacillus* was identified (r > 0.7, p < 0.05) in the ER-negative group. Significant correlations were observed for phospholipase A2 group IIE (PLA2G2E, Table 3), and the expression of 219 other genes including 6 keratin-associated protein genes was correlated with the activity of *Lactobacillus*. Moreover, the expression of 258 genes was negatively correlated with the activity of *Lactobacillus* (r < -0.7, p < 0.05).

**Table 3:** Gene expression positively correlated to Lactobacillus abundance and negatively correlated to Bacteroides abundance in ER-negative tumor tissues (r > 0.7 and *p* < 0.05)

Gene Symbol	Taxonomy 1	Correlation Coefficient	*P* value	Taxonomy 2	Correlation Coefficient	*P* value	Description
IL5RA				Lactobacillus	0.92	0.000001	interleukin 5 receptor, alpha
VN1R66P				Lactobacillus	0.92	0.000001	vomeronasal 1 receptor 66 pseudogene
KRTAP10-2				Lactobacillus	0.91	0.000002	keratin associated protein 10 − 2
CCDC34	Bacteroides	-0.92	0.000001	Lactobacillus	0.89	0.000009	coiled-coil domain containing 34
C1orf68				Lactobacillus	0.89	0.000012	chromosome 1 open reading frame 68
BRD1	Bacteroides	-0.85	0.000073	Lactobacillus	0.85	0.00005	bromodomain containing 1
TRGVB	Bacteroides	-0.89	0.000009	Lactobacillus	0.85	0.000057	T-cell receptor gamma variable B (pseudogene)
OGFOD2				Lactobacillus	0.84	0.000076	2-oxoglutarate and iron-dependent oxygenase domain containing 2
PRCC	Bacteroides	-0.84	0.000096	Lactobacillus	0.84	0.000082	papillary renal cell carcinoma (translocation-associated)
TBL2				Lactobacillus	0.84	0.000086	actin, beta-like 2
ANAPC4				Lactobacillus	0.84	0.000104	anaphase promoting complex subunit 4
SIRPD				Lactobacillus	0.83	0.000121	signal-regulatory protein delta
**PLA2G2E**	**Bacteroides**	**-0.77**	**0.000821**	**Lactobacillus**	**0.83**	**0.000135**	**phospholipase A2, group IIE**
MALL				Lactobacillus	0.83	0.000143	mal, T-cell differentiation protein-like
ERC1				Lactobacillus	0.83	0.000147	ELKS/RAB6-interacting/CAST family member 1
PYCR1				Lactobacillus	0.82	0.000163	pyrroline-5-carboxylate reductase 1
CGB1				Lactobacillus	0.82	0.000205	chorionic gonadotropin, beta polypeptide 1
KRTAP5-8				Lactobacillus	0.81	0.000228	keratin associated protein 5–8
**HIST2H2BE**	**Bacteroides**	**-0.94**	**0.0000002**	**Lactobacillus**	**0.80**	**0.000357**	**histone cluster 2, H2be**
**CRKL**	**Bacteroides**	**-0.85**	**0.000050**	**Lactobacillus**	**0.76**	**0.000989**	**v-crk avian sarcoma virus CT10 oncogene homolog-like**
**CCND1**	**Bacteroides**	**-0.84**	**0.000080**	**Lactobacillus**	**0.76**	**0.000935**	**cyclin D1**
CDC42	Bacteroides	-0.72	0.002428	Lactobacillus	0.72	0.002575	cell division control protein 42 homolog
LTBR	Bacteroides	-0.71	0.002741	Lactobacillus	0.74	0.001720	lymphotoxin beta receptor (TNFR superfamily, member 3)
HIST2H2BF	Bacteroides	-0.71	0.002930	Lactobacillus	0.70	0.003442	histone cluster 2, H2bf
SHC1	Bacteroides	-0.87	0.00026	Lactobacillus	0.71	0.003305	SHC (Src homology 2 domain containing) transforming protein 1

Seven enriched KEGG pathways were correlated with the activity of *Lactobacillus* in the ER-negative group (p < 0.05), including “renal cell carcinoma” (CDC42, CRKL, PRCC), “viral carcinogenesis” (LTBR, CCND1, HIST2H2BE, CDC42, HIST2H2BF) and 94 GO terms were correlated with the activity of *Lactobacillus* in this group (p < 0.05) as presented in Supplementary file 6 A-B [22].

### Correlation analysis between gene expression and the activity of *Bacteroides* in the ER-negative tumors

A significant correlation between gene expression and the relative activity of *Bacteroide* was not found among 55 ER-positive samples. However, significantly lower expression of several genes, including PLA2G2E, HIST2H2BE, SHC1, CRKL, CCND1, PRCC, CDC42 and LTBR, was correlated with the relative activity of *Bacteroides* in 15 ER-negative samples (Table 3). The expression of CASP2 positively correlated with the relative activity of *Bacteroides* and *Alloprevotella* (correlation coefficient score = 0.79, p < 0.01).

## Discussion

### Microbiota characteristics of ER-negative tumor tissue and the possible reasons

Whether the microorganisms in FFPE tissues may be arise from environmental contamnation rather than originate from tissue was clear after comparison with the environmental control samples. The analysis of 16 S RNA sequencing of the solvent used in this study showed completely different microbiota content than that in FFPE samples. The analysis of ITS RNA sequencing of the environmental control failed in amplification indicating no fungi was detected. In addition, paraffin can preserve microorganisms, but microorganisms will not proliferate in paraffin. Therefore, the results regarding bacteria and fungi obtained from our artificial results are convincing.

The characteristics of the microbiota of breast tumor tissue have been documented in European, Canadian and American studies. The principal phylum in healthy breast tissue and tissue adjacent to breast tumors is *Proteobacteria* which exceeds 50% abundance, followed by *Firmicutes* (35%), *Actinobacteria* (10%), and *Bacteroidetes* (5%), as revealed by Canadian and Irish studies [11]. The breast tumor tissues have higher relative abundance of *Protebacteria* than healthy tissues shown by Italian study [23]. In non-Hispanic White women and non-Hispanic Black women, *Proteobacteria* is most abundant in normal, normal adjacent to tumor, and breast tumor tissue, with fewer *Firmicutes*, *Bacteroidetes* and *Actinobacteria* [24]. Similar results were shown for USA women with more than 50% relative abundance of *Proteobacteria* followed by *Firmicutes*, *Actinobacteria* and *Bacteroidetes* in healthy, high risk, tumor adjacent and tumor tissues [25]. These studies indicate similar results for Canadian, Irish, Italian, American, non-Hispanic White women and non-Hispanic Black women in microbiota phyla distribution, but there are significant differences in microbiota genera distribution as described in introduction. There are might be significant differences of breast tissue microbiota among ethnic groups. On the other hand, distinct microbial signatures associate with different breast cancer types, including higher abundance of genus *Lactobacillus* in ER+/HER2- ER+/HER2 + and ER-/HER2 + breast tumor than healthy breast samples, and in triple negative tumors several genera significantly correlate with severe (dead) clinical outcomes [26]. In this study, the microbial characteristics of 70 breast tumors of Asian women were analyzed. Although the top phyla were *Bacteroidetes, Firmicutes, Actinobacteria* and *Proteobacteria* as same as previous studies, the distribution was unique in this study especially for ER negative tumors in which there were higher activity of genus *Lactobacillus* (phylum *Firmicutes*) and positively correlated with several carcinogenesis genes expression. There are might be relationships between breast tissue microbes and cancer development. Because of the limited FFPE samples, in this study, total RNA was reverse transcribed into cDNA and used for both of PCR amplification of the bacterial 16 S rRNA and human gene microaary hybridization. Higher *Bacteroidetes* and *Firmicutes* activity might indicate the higher transcriptional activity but not always represent higher abundance in tumor tissues.

*Lactobacillus* was not found in the milk of healthy human [27], indicating that infection by this genus was more likely to occur from the outside environment. The hypothesis for the microbiota characteristics of triple-negative breast tumor tissue is that during lactation, *Lactobacillus* invades the mother’s nipple and breast from baby’s mouth because infants may inhale the bacteria in the birth canal [28]. In nipple aspirate fluid collected from patients with breast cancer, there is a relatively higher activity of the genus *Alistipes* (*Bacteroidetes*) than in the fluid from healthy control women [29]. This finding is consistent with the results in this study.

### The tumor microbiota correlated with tumorgenesis gene expression in ER-negative breast cancer

It has been reported that the bacterial DNA load in breast tumors correlates inversely with advanced cancer [30], but few studies have examined the relationship between the tumor microbiota and cellular gene expression. To determine whether the change in the microbiota in breast cancer is related to the occurrence and development of cancer, we analyzed the correlation between the microbiota and gene expression using the same total RNA extracted from FFPE samples. There was no correlation between the bacterium and gene expression in ER-positive (ER+/HER2- and ER+/HER2 + types) tumor tissue. The expression of 221 genes was positively associated with the higher activity of *Lactobacillus* in ER-negative (ER-/HER2 + and triple-negative types) samples (r > 0.7, p < 0.05) including breast cancer biomarker genes PLA2G2E, HIST2H2BE, CRKL, and CCND1 (Table 3) and the expression of same genes was negatively correlated with the relative activity of *Bacteroides* (Table 3). Fungal activity was not related to any gene expression in the same ER-negative samples.

PLA2G2E (group IIE sPLA2) expression was significantly associated with the activity of *Lactobacillus* in the ER-negative group, and its expression was 1.21-fold higher in the ER-negative group than in the ER-positive group (p < 0.001). PLA2s comprise a superfamily that is generally divided into six subfamilies: cytosolic PLA2s (cPLA2s), calcium-independent PLA2s (iPLA2s), secreted PLA2s (sPLA2s), lysosomal PLA2s, platelet-activating factor acetylhydrolases, and adipose-specific PLA2s [31]. PLA2 is an esterase that cleaves glycerophospholipids to release fatty acids and lysophospholipids and may be associated with tumorigenesis in human tissues [32]. An analysis of receiver operating characteristic curves revealed that plasma PLA2 (sPLA2s) activities were higher in patients with breast cancer than in healthy controls. Plasma PLA2 activity may serve as a biomarker for patients with breast cancer [33].

CRKL (Crk-like) is an adapter protein that has crucial roles in multiple biological processes, including cell proliferation, adhesion, and migration. CRKL induces cyclin D1 and phosphorylated extracellular signal-regulated kinase expression, overexpression of CRKL correlates with progression and malignant proliferation of human breast cancers [34]. CCND1 (cyclin D1) has been solidly established as an oncogene with an important pathogenetic role in breast cancer [35]. CCND1 overexpression is found in more than 50% of human breast cancers and causes mammary cancer in transgenic mice. Dysregulation of CCND1 gene expression or function contributes to the loss of normal cell cycle control during tumorigenesis [36]. HIST2H2BE (histone cluster 2) promotes the progression of invasive ductal carcinoma [37].

Specific microbes such as *Bacteroides*, *Streptococcus*, *Bacteroides massiliensis*, *Faecalibacterium prausnitzii*, *Eubacterium rectalie*, and *Mycoplasma genitalium* have been associated with differing risks of prostate cancer development or the extensiveness of prostate cancer disease [38]. Moreover, the present study found an association of the microbiota in ER-negative breast tumor with breast cancer biomarker gene expression which may be associated with tumorigenesis.

The tumor microbiota disorder (higher *Lactobacillus* activity) in triple-negative breast cancer, possibly caused by external factors, associated with the carcinogenesis gene expression. Although the mechanism by which the tumor microbiota relating to the gene expression remains unknown, we speculate that the breast tumor microenvironment might be involved in some way. However, these results have limitations for the smaller sample size especially of the triple-negative group including only 7 samples, and need to be confirmed in a larger cohort. Whether the disorder of the microbiota in breast tissue is related to the lactation history of spontaneously delivered infants and mothers’ vaginitis also remains to be confirmed.

This was the first study to identify a significant association of the breast tumor tissue microbiota with the expression of carcinogenesis genes in ER-negative breast cancer. This finding indicated that changes in the microbiota of breast tissue induced by external factors might be one of the key cause of breast carcinogenesis. We believe that the results in this study provide new targets for breast cancer treatment.

## Electronic supplementary material

Below is the link to the electronic supplementary material.

Supplementary Material 1

## Data Availability

The microarray data were deposited in the Gene Expression Omnibus (GEO) database under accession code GSE183231 and GSE185439, which were obtained from the same cohort experiment. The 16 S rRNA and ITS sequencing data have been uploaded to GenBank Sequence Read Archive under accession number PRJNA769523. The codes used during the study are available from the corresponding author by request, including program codes for Figs. 1 and 2.
