# Supplementary material for: Relationship between tumor microbiota transcriptional activity and gene expression in breast cancer
Source: BMC Cancer. 2023 Mar 16;23:252. doi: 10.1186/s12885-023-10726-4 (PMC10018882; doi:10.1186/s12885-023-10726-4)
Supplement: Supplementary file 1 — Supplementary Material 1 [file 12885_2023_10726_MOESM1_ESM.pdf]

Relationship between tumor microbiota transcriptional activity and gene expression in breast cancer

Funning title: Tumor microbiota relates to carcinogenesis

Enuo Liu[1,2], Fan Zhang[4], Tiansheng Xu[4], Luyi Ye[1], Sean Si Qian Ma[1], Zai-Si Ji[1,3*]

[1] NHC Key Laboratory of Reproduction Regulation (Shanghai Institute for Biomedical and Pharmaceutical Technologies), Fudan University, 2140 Xietu road, Shanghai 200032, China

[2] College of Food Sciences and Technology, Shanghai Ocean University, 999 Hucheng Road, Shanghai, China

[3] Division of Research and Development, Meiji Co., Ltd, 1-29-1 Nanakuni, Hachiouji, Tokyo 192-0919, Japan

[4] Shanghai OE Biotech Co., Ltd, 1188 Lianhang road, Shanghai, 201114, China

[*] for Correspondence: Zai-Si Ji, jizaisi@sibpt.com; ORCID 0000-0002-6038-1517

Enuo Liu: liuenuo@sibpt.com

Fan Zhang: fan.zhang@oebiotech.com

Tiansheng Xu: tiansheng.xu@oebiotech.com

Luyi Ye: yeluyi@sibpt.com

Sean Si Qian Ma: seansiqianma@hotmail.com

Supplementary file 1 The relative activity of bacteria and fungi distribution in environmental controls and tumor groups. A. the microbial genuses distribution in environmental controls; B. the microbial genuses distribution in four tumor groups; C. and the photo of amplification step of ITS1 variable region in two environmental controls.

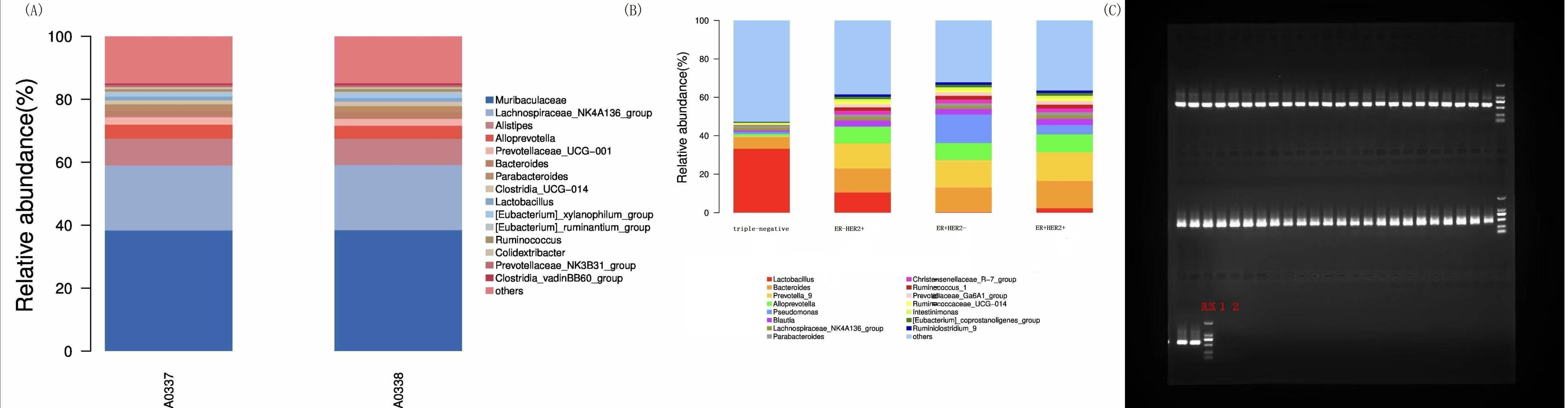
(A)
Relative abundance(%)
100
80
60
40
20
0
A0337
A0338
Muribaculaceae
Lachnospiraceae_NK4A136_group
Alistipes
Alloprevotella
Prevotellaceae_UCG-001
Bacteroides
Parabacteroides
Clostridia_UCG-014
Lactobacillus
[Eubacterium]_xylanophilum_group
[Eubacterium]_ruminantium_group
Ruminococcus
Colidextribacter
Prevotellaceae_NK3B31_group
Clostridia_vadinBB60_group
others
(B)
Relative abundance(%)
100
80
60
40
20
0
triple-negative
ER-HER2+
ER+HER2-
ER+HER2+
Lactobacillus
Bacteroides
Prevotella_9
Alloprevotella
Pseudomonas
Blautia
Lachnospiraceae_NK4A136_group
Parabacteroides
Christensenellaceae_R-7_group
Ruminococcus_1
Prevotellaceae_Ga6A1_group
Ruminococcaceae_UCG-014
Intestinimonas
[Eubacterium]_coprostanoligenes_group
Ruminiclostridium_9
others
(C)
DL2K 1 2

Supplementary file 2 Analysis of fungal profiles in four groups. A. Good's coverage for the four groups; B. comparison of fungal richness (Chao 1) among the four groups; C. comparison of fungal diversity (Shannon) among the four groups; D. analysis of beta diversity (NMDS) among the four groups. Wilcoxon rank sum test was used for comparisons. * p value <0.05, ** p value < 0.01, *** p value <0.001.

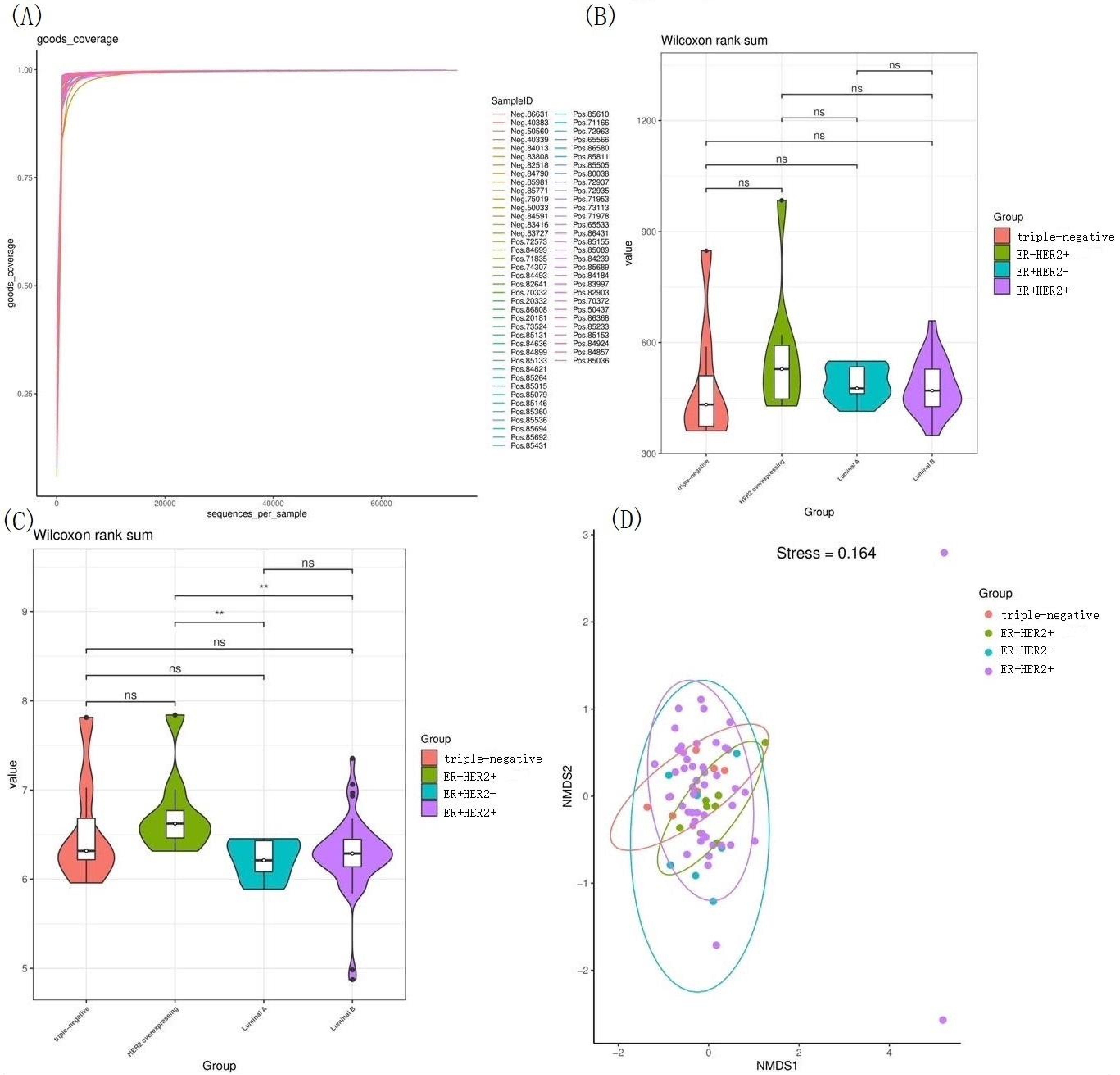
(A)
goods_coverage
goods_coverage
sequences_per_sample
1.00
0.75
0.50
0.25
0
20000
40000
60000
SampleID
Neg.86631
Neg.40383
Neg.50560
Neg.40339
Neg.84013
Neg.83808
Neg.82518
Neg.84790
Neg.85981
Neg.85771
Neg.75019
Neg.50033
Neg.84591
Neg.83416
Neg.83727
Pos.72573
Pos.84699
Pos.71835
Pos.74307
Pos.84493
Pos.82641
Pos.70332
Pos.20332
Pos.86808
Pos.20181
Pos.73524
Pos.85131
Pos.84636
Pos.84899
Pos.85133
Pos.84821
Pos.85264
Pos.85315
Pos.85079
Pos.85146
Pos.85360
Pos.85536
Pos.85694
Pos.85692
Pos.85431
Pos.85610
Pos.71166
Pos.72963
Pos.65566
Pos.86580
Pos.85811
Pos.85505
Pos.80038
Pos.72937
Pos.72935
Pos.71953
Pos.73113
Pos.71978
Pos.65533
Pos.86431
Pos.85155
Pos.85089
Pos.84239
Pos.85689
Pos.84184
Pos.83997
Pos.82903
Pos.70372
Pos.50437
Pos.86368
Pos.85233
Pos.85153
Pos.84924
Pos.84857
Pos.85036
(B)
Wilcoxon rank sum
value
Group
300
600
900
1200
ns
ns
ns
ns
ns
ns
triple-negative
HER2 overexpressing
Luminal A
Luminal B
Group
triple-negative
ER-HER2+
ER+HER2-
ER+HER2+
(C)
Wilcoxon rank sum
value
Group
5
6
7
8
9
ns
**
**
ns
ns
ns
triple-negative
HER2 overexpressing
Luminal A
Luminal B
Group
triple-negative
ER-HER2+
ER+HER2-
ER+HER2+
(D)
Stress = 0.164
NMDS2
NMDS1
3
2
1
0
−1
−2
−2
0
2
4
Group
triple-negative
ER-HER2+
ER+HER2-
ER+HER2+

Supplementary file 3 Mean relative activity of top 4 fungal phyla (mean relative activity >0.01) identified in four groups.

Taxon	*p* value	FDR *p*	Mean of relative activity<br>ER+/HER2-	ER+/HER2+	ER-/HER2+	Triple-negative
Ascomycota	0.2624	0.6123	0.3388	0.3825	0.4039	0.4088
Basidiomycota	0.0268	0.1252	0.2599	0.1742	0.2044	0.1424
Rozellomycota	0.3563	0.6949	0.0169	0.0211	0.0170	0.0299
Mortierellomycota	0.4598	0.6949	0.0223	0.0311	0.0325	0.0204

P value and FDR (false-discovery rate) *p* value were determined by ANOVA statistical test.

Supplementary file 4 Analysis of differentially expressed genes between ER negative and ER positive samples of breast cancer patients. A. the volcano plot of differentially expressed genes; B. coding, non-coding and other genes in differently expressed genes; C. top 20 of enriched GO terms of differentially expressed genes.

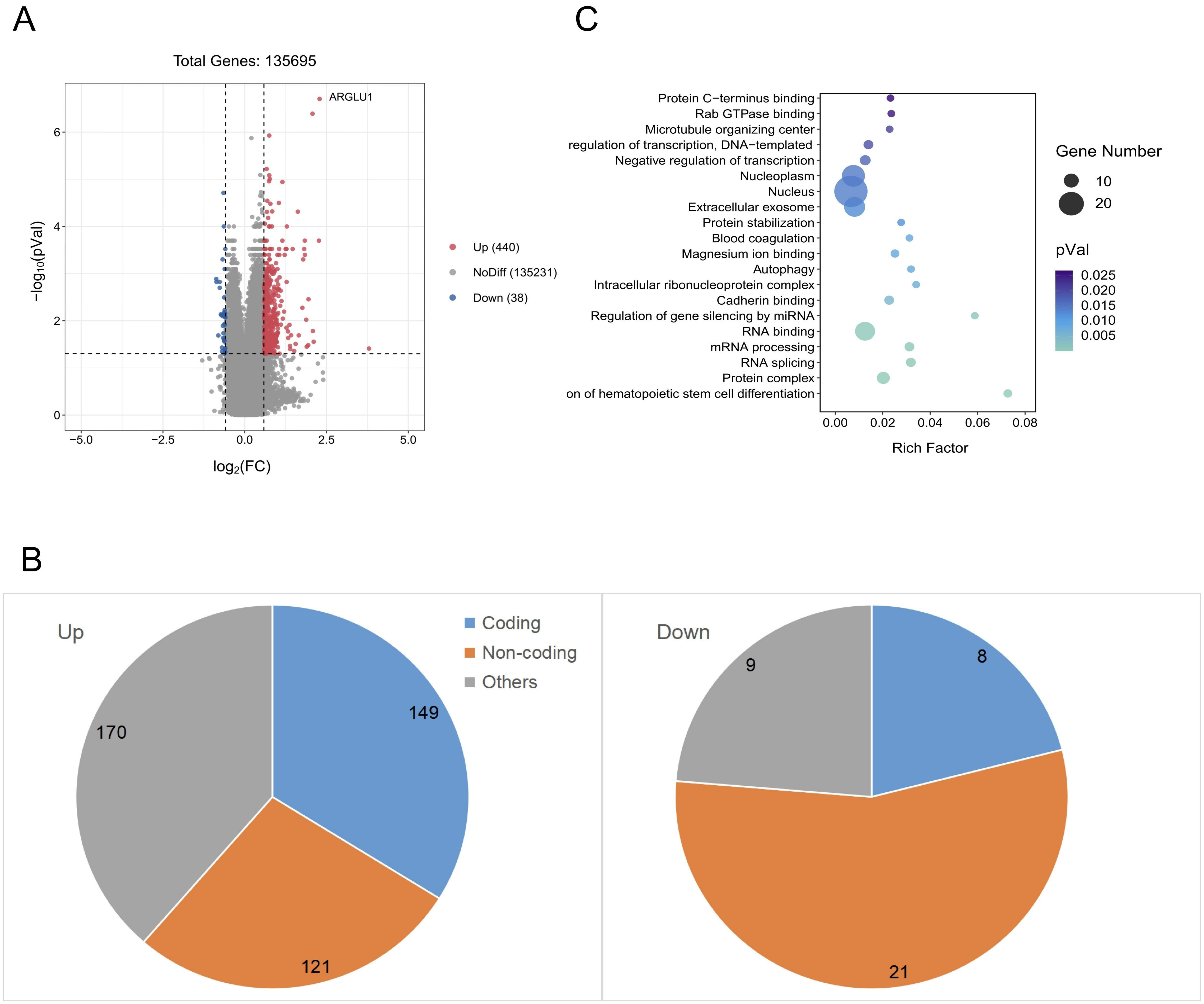
A
Total Genes: 135695
ARGLU1
−log10(pVal)
log2(FC)
Up (440)
NoDiff (135231)
Down (38)
C
Protein C−terminus binding
Rab GTPase binding
Microtubule organizing center
regulation of transcription, DNA−templated
Negative regulation of transcription
Nucleoplasm
Nucleus
Extracellular exosome
Protein stabilization
Blood coagulation
Magnesium ion binding
Autophagy
Intracellular ribonucleoprotein complex
Cadherin binding
Regulation of gene silencing by miRNA
RNA binding
mRNA processing
RNA splicing
Protein complex
on of hematopoietic stem cell differentiation
Rich Factor
Gene Number
10
20
pVal
0.025
0.020
0.015
0.010
0.005
B
Up
Coding
Non-coding
Others
149
121
170
Down
8
21
9

Supplementary file 5 KEGG Pathway enrichment in ER negative samples vs ER positive samples (p value <0.05).

Term_description	List Hits	Population Hits	Enrichment score	p-value	Gene Symbols
One carbon pool by folate	2	20	24.99	0.003	MTHFD1;GART
Basal transcription factors	2	44	11.36	0.013	TAF11;MNAT1
Autophagy - animal	3	128	5.86	0.014	HIF1A;VMP1;HMGB1
Vitamin B6 metabolism	1	6	41.64	0.024	PDXK
Purine metabolism	3	174	4.31	0.031	POLR2J3;PNPT1;GART
Platinum drug resistance	2	71	7.04	0.032	MGST1;MSH6

Supplementary file 6 Analysis of genes expression correlated to Lactobacillus abundance in ER negative tumor tissues. A. top 20 of entiched GO terms; B. top 20 of enriched KEGG pathways.

A

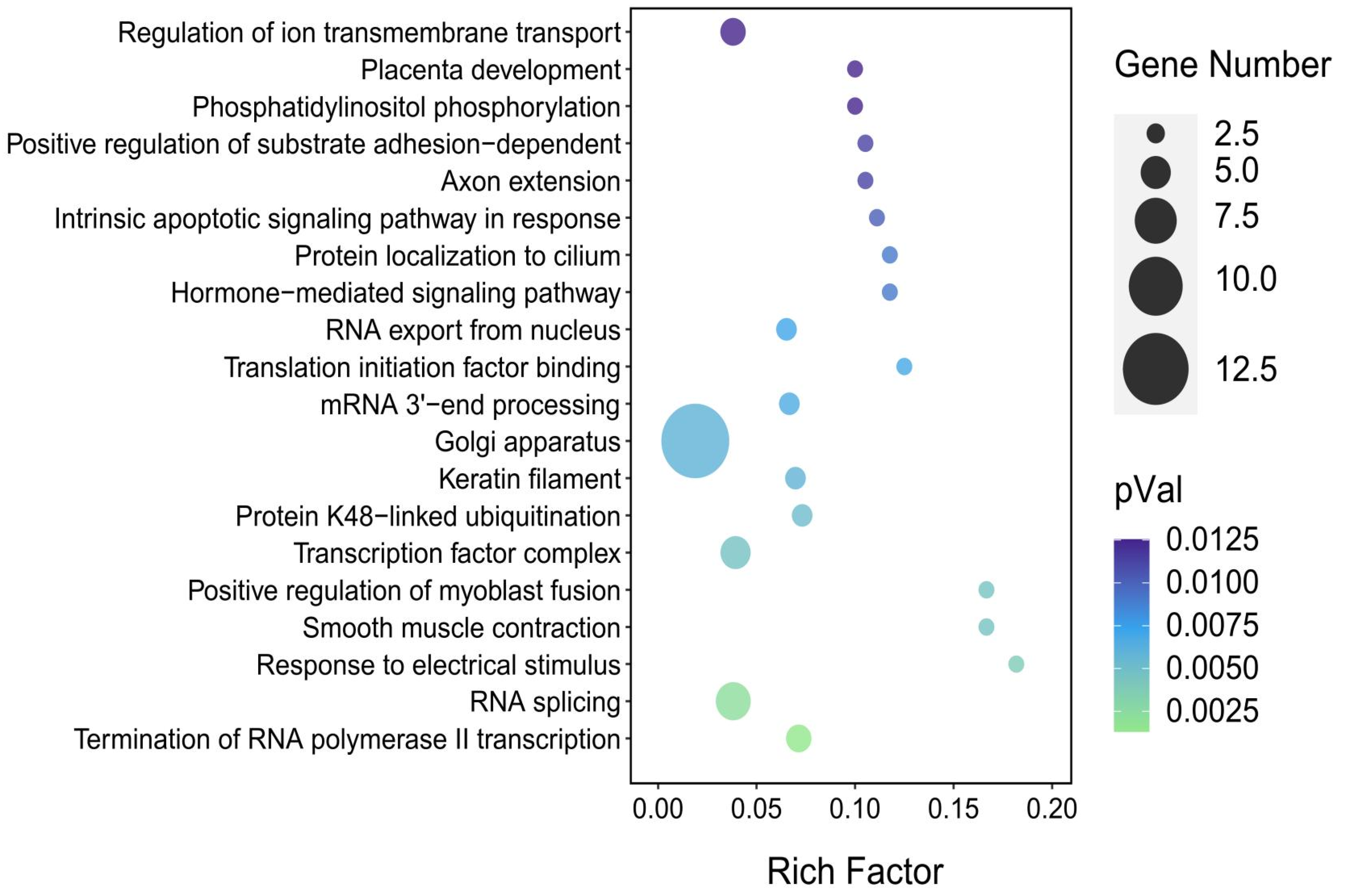
Regulation of ion transmembrane transport
Placenta development
Phosphatidylinositol phosphorylation
Positive regulation of substrate adhesion−dependent
Axon extension
Intrinsic apoptotic signaling pathway in response
Protein localization to cilium
Hormone−mediated signaling pathway
RNA export from nucleus
Translation initiation factor binding
mRNA 3'−end processing
Golgi apparatus
Keratin filament
Protein K48−linked ubiquitination
Transcription factor complex
Positive regulation of myoblast fusion
Smooth muscle contraction
Response to electrical stimulus
RNA splicing
Termination of RNA polymerase II transcription
0.00 0.05 0.10 0.15 0.20
Rich Factor
Gene Number
2.5
5.0
7.5
10.0
12.5
pVal
0.0125
0.0100
0.0075
0.0050
0.0025

B

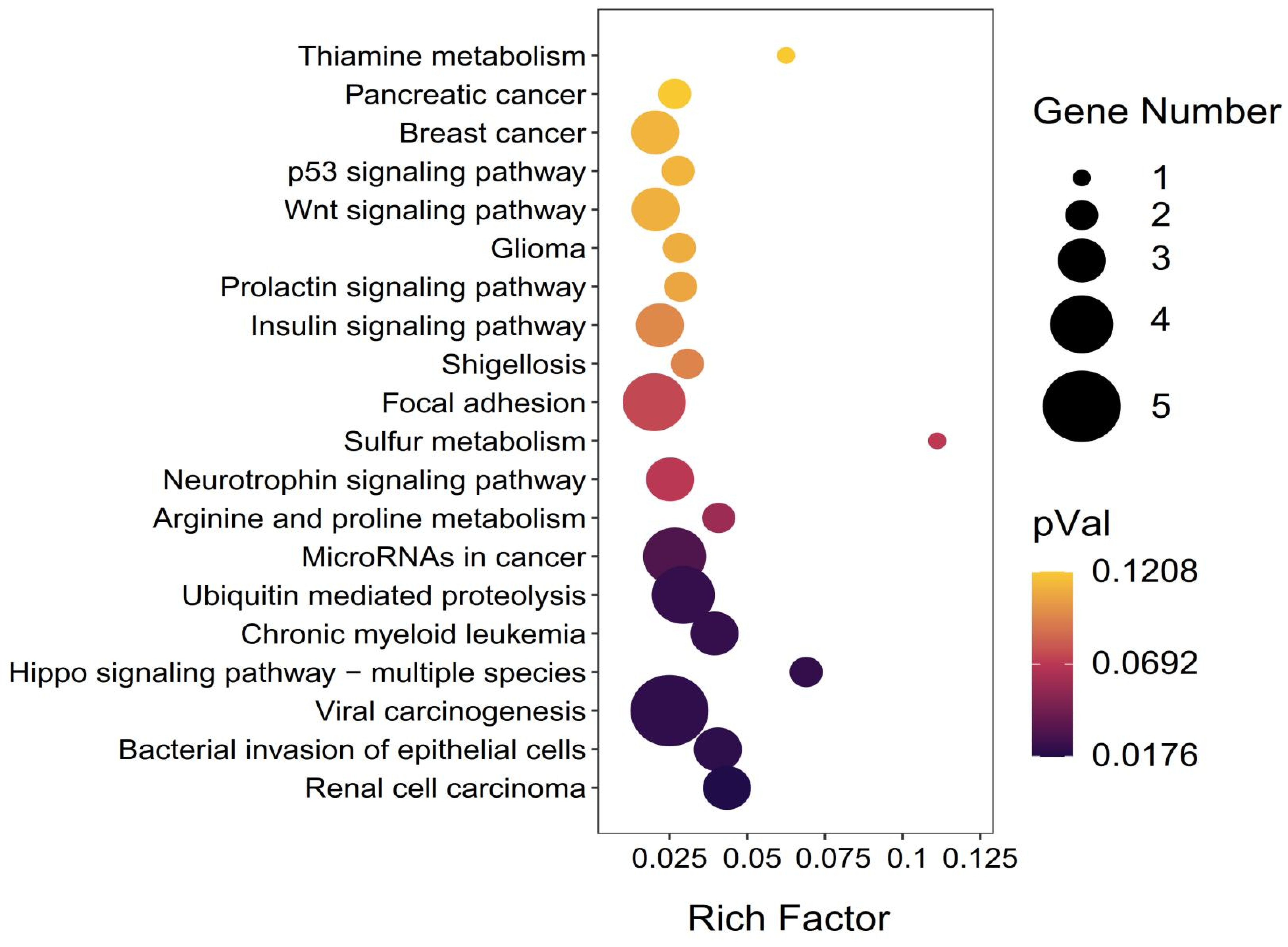
Thiamine metabolism
Pancreatic cancer
Breast cancer
p53 signaling pathway
Wnt signaling pathway
Glioma
Prolactin signaling pathway
Insulin signaling pathway
Shigellosis
Focal adhesion
Sulfur metabolism
Neurotrophin signaling pathway
Arginine and proline metabolism
MicroRNAs in cancer
Ubiquitin mediated proteolysis
Chronic myeloid leukemia
Hippo signaling pathway − multiple species
Viral carcinogenesis
Bacterial invasion of epithelial cells
Renal cell carcinoma
0.025 0.05 0.075 0.1 0.125
Rich Factor
Gene Number
1
2
3
4
5
pVal
0.1208
0.0692
0.0176
